# Sex differences in the neuroanatomy of alcohol dependence: hippocampus and amygdala subregions in a sample of 966 people from the ENIGMA Addiction Working Group

**DOI:** 10.1038/s41398-021-01204-1

**Published:** 2021-03-04

**Authors:** Sally Grace, Maria Gloria Rossetti, Nicholas Allen, Albert Batalla, Marcella Bellani, Paolo Brambilla, Yann Chye, Janna Cousijn, Anna E Goudriaan, Robert Hester, Kent Hutchison, Izelle Labuschagne, Reza Momenan, Rocio Martin-Santos, Peter Rendell, Nadia Solowij, Rajita Sinha, Chiang-shan Ray Li, Lianne Schmaal, Zsuzsika Sjoerds, Chao Suo, Gill Terrett, Ruth J. van Holst, Dick J. Veltman, Murat Yücel, Paul Thompson, Patricia Conrod, Scott Mackey, Hugh Garavan, Valentina Lorenzetti

**Affiliations:** 1 Neuroscience of Addiction & Mental Health Program, Healthy Brain and Mind Research Centre, School of Behavioural & Health Sciences, Faculty of Health Sciences, Australian Catholic University, Melbourne, VIC Australia; 2 Department of Neurosciences, Biomedicine and Movement Sciences, Section of Psychiatry, University of Verona, Verona, Italy; 3 Department of Neurosciences and Mental Health, Fondazione IRCCS Ca’ Granda Ospedale Maggiore Policlinico, Milan, Italy; 4 Department of Psychology, University of Oregon, Eugene, OR USA; 5 Department of Psychiatry, UMC Utrecht Brain Center, Utrecht University, Utrecht, Netherlands; 6 Department of Pathophysiology and Transplantation, University of Milan, Milan, Italy; 7 Turner Institute for Brain and Mental Health, School of Psychological Sciences, Monash University, Clayton, VIC Australia; 8 Department of Psychology, University of Amsterdam, Amsterdam, the Netherlands; 9 Department of Psychiatry, Amsterdam Institute for Addiction Research and Amsterdam Neuroscience Research Institute, Amsterdam UMC, University of Amsterdam, Amsterdam, the Netherlands; 10 School of Psychological Sciences, University of Melbourne, Melbourne, Australia; 11 Department of Psychology and Neuroscience, University of Colorado Boulder, Boulder, CO USA; 12 Clinical NeuroImaging Research Core, Office of the Clinical Director, National Institute on Alcohol Abuse and Alcoholism, Bethesda, MD USA; 13 Department of Neurosciences and Behavior, Ribeirão Preto Medical School, University of São Paulo, Av. Bandeirantes, Ribeirão Preto, São Paulo Brazil; 14 School of Psychology and Illawarra Health and Medical Research Institute, University of Wollongong, Wollongong, Australia; 15 Department of Psychiatry, Yale University School of Medicine, New Haven, CT USA; 16 Orygen, The National Centre of Excellence in Youth Mental Health, Parkville, Australia; 17 Centre for Youth Mental Health, The University of Melbourne, Melbourne, VIC 3052 Australia; 18 Institute of Psychology. Cognitive Psychology Unit, Leiden University, Leiden, The Netherlands; 19 Departments of Neurology, Radiology, Engineering, & Psychiatry, Imaging Genetics Center, Mark & Mary Stevens Institute for Neuroimaging & Informatics, Keck School of Medicine, University of Southern California, Marina del Rey, CA USA; 20 Department of Psychiatry, Université de Montreal, CHU Ste Justine Hospital, Montreal, QC Canada; 21 Departments of Psychiatry and Psychology, University of Vermont, Burlington, VT USA

**Keywords:** Neuroscience, Psychiatric disorders

## Abstract

Males and females with alcohol dependence have distinct mental health and cognitive problems. Animal models of addiction postulate that the underlying neurobiological mechanisms are partially distinct, but there is little evidence of sex differences in humans with alcohol dependence as most neuroimaging studies have been conducted in males. We examined hippocampal and amygdala subregions in a large sample of 966 people from the ENIGMA Addiction Working Group. This comprised 643 people with alcohol dependence (225 females), and a comparison group of 323 people without alcohol dependence (98 females). Males with alcohol dependence had smaller volumes of the total amygdala and its basolateral nucleus than male controls, that exacerbated with alcohol dose. Alcohol dependence was also associated with smaller volumes of the hippocampus and its CA1 and subiculum subfield volumes in both males and females. In summary, hippocampal and amygdalar subregions may be sensitive to both shared and distinct mechanisms in alcohol-dependent males and females.

## Introduction

The burden of alcohol dependence to society is substantial, with an estimated global annual economic impact of between 210 and 665 billion US dollars arising from health consequences, workplace issues, safety, drink-driving, and criminal costs^1,2^. Alcohol dependence is more common in men than in women^3^. However, the impact of alcohol dependence on women is particularly problematic as women are more likely to suffer negative consequences of drinking, and have shown an increase in recent years in their rates of alcohol dependence^4^. Known as the telescoping effect^3^ women who drink excessively on average have worse medical and mental health outcomes^5^, transition to dependence more quickly^6^, have stronger cravings^7^, and find it harder to quit drinking^8^, compared to their male counterparts. These differences may arise from distinct neurobiological mechanisms in men and women with alcohol dependence.

Current neurobiological theories of addiction emphasise brain systems underlying motivation, learning, and stress (e.g., amygdala and hippocampus), which are implicated in compulsive alcohol use, alcohol-seeking, and high-stress levels while craving alcohol^9^. However, there is a knowledge gap in the evidence informing such theories as they are largely biased by the inclusion of male-only samples, and three-quarters of published studies have failed to evaluate the interaction between alcohol dependence and sex^10,11^. International and national drug agencies recognise that addressing this gap is now a priority (e.g., U.S. National Institute of Health and the U.S. Federal Drug Administration)^12^. New evidence on sex differences within the neurobiology of alcohol dependence is required to advance existing neuroscientific theories of addiction, clarify the role of sex in the mechanisms of substance use disorders, contribute to the design of gender-tailored treatments, and inform personalised medicine.

Animal models of alcohol dependence highlight a key role of select subregions within the amygdala and hippocampus in stress, learning, and memory processes crucial for developing and maintaining addiction^13^. Within the amygdala, dendritic spine remodelling and glutamate function of the central nucleus are implicated in the drive for using alcohol^9^ and anxiety during withdrawal^14^ in alcohol-dependent rats. The firing of neurons within the basolateral amygdala is also involved in reward learning and the conditioned-craving for alcohol^9,15^, and alcohol-related negative affect during withdrawal^13,16^. Additionally, sex differences in the integrity of select amygdala nuclei in alcohol administration studies are emerging. For example, basolateral and central amygdala neurons of female compared to male rats show enhanced neuronal responses to stress during ethanol self-administration, and may therefore be implicated in stress-induced alcohol seeking^14^. Further, glutamate neurons within the central amygdala in male compared to female animals show greater sensitivity to ethanol, and this may contribute to the higher propensity toward alcohol use observed in males^14^.

In relation to the hippocampus, alterations in specific subfields are implicated in alcohol dependence, intoxication, and withdrawal^17–21^. For example, chronic exposure to alcohol leads to the loss of pyramidal and granule cells of the dentate gyrus, CA1, and CA3^22,23^, and to reduced neurogenesis within the dentate gyrus^22^. Early findings in alcohol-dependent animals also show sex differences in hippocampal subfields. Alcohol dependence and increased drug-seeking are linked to ultrastructural damage and aberrant neural activity of select hippocampus subfields in female but not male rats (e.g., dentate gyrus, CA1, and CA3)^22–24^. These structural damages are associated with a range of adverse outcomes characteristic of alcohol dependence: including spatial learning and memory deficits^17,25–27^, increased alcohol taking, and relapse^20^.

The above-mentioned findings on subregional alterations and sex differences in alcohol dependence are yet to be corroborated in humans. Smaller volumes of the whole amygdala^28–30^ and hippocampus^31–35^ have been identified in people with alcohol dependence compared to controls. However, the neuroimaging evidence to date in people with alcohol dependence has focussed on mostly male samples and tends not to examine or report interactions with sex^11^. Preliminary evidence of sex-related effects in alcohol dependence show smaller total grey and white matter volumes^3,36,37^ and smaller total volumes of the hippocampus^34^ in alcohol-dependent females compared to male counterparts, despite equal or lesser alcohol use. There is also evidence of volume loss within specific subregions of the amygdala (i.e., basolateral) and the hippocampus (i.e., subiculum) in alcohol dependence^38^. However, whether such subregional effects are driven by either males or females, or by each sex equally, has not been examined. Methodological limitations may account for this knowledge gap, such as the use of small sample sizes with insufficient power to concurrently examine the effects of sex and alcohol on brain volumes, and the limited resolution and accuracy of traditional neuroimaging data analysis methods to segment amygdala and hippocampus subregions^39^. Global consortia comprising large population-representative samples, together with the recent release of new and reliable algorithms to segment subregions^40^, provide a unique opportunity to examine in detail amygdala and hippocampus subregions in males and females with alcohol dependence.

We aimed to validate animal findings in a sample of 966 human participants selected from the ENIGMA Addiction Working Group (https://www.enigmaaddictionconsortium.com/). The sample comprised 323 people without alcohol dependence (225 males and 98 females) and a large sample of 643 people with alcohol dependence (418 males and 225 females). We hypothesised that: (i) on average, people with alcohol dependence *versus* controls would show smaller volumes of specific amygdala nuclei (i.e., central and basolateral)^19–22^ and hippocampal subfields (i.e., CA1, CA3, dentate gyrus and subiculum)^27–32^; (ii) females with alcohol dependence would have smaller volumes than female controls and males with alcohol dependence^3,34,36,37,41–44^; (iii) a greater number of monthly standard alcohol drinks would predict lower volumes in the hypothesised subregions in both sexes^3,36,37^. Given the opportunities afforded by the large sample of this study, we also explored group and group-by-sex differences in additional amygdala nuclei (i.e., anterior amygdala area, cortico-amygdaloid transition area, paralaminar, accessory basal, medial, and cortical nuclei) and hippocampal subfields (i.e., CA4, hippocampal-amygdaloid transition area, fimbria, fissure, tail, presubiculum, and parasubiculum).

## Materials and methods

### Participants

This study was pre-registered on the *Open Science Framework* in September 2018 (see https://osf.io/gz96w). Demographic, neuroimaging, and substance use (alcohol and tobacco) data were collected for 1,325 participants from 10 research sites that are part of the ENIGMA Addiction Working Group (https://www.enigmaaddictionconsortium.com/). Supplementary Table 1 provides summary information for each site: including site location, study inclusion/exclusion criteria, MRI scanner type, acquisition sequences and instruments used to measure substance use. We screened the original sample using the following exclusion criteria: (i) lifetime and/or current primary psychiatric disorders other than alcohol dependence and/or current dependence on substances other than alcohol (*n* = 125 alcohol group); (ii) abstinence > 30 days (*n* = 15 alcohol group); (iii) IQ < 80 (*n* = 15 alcohol group); (iv) missing data for key variables including sex (*n* = 68 alcohol group) and years of education (*n* = 45 alcohol group and *n* = 73 controls); and (v) MRI artefacts that undermined the validity of the volumes (*n* = 15 alcohol group, and *n* = 3 controls). The final sample comprised 966 participants aged on average 32.47 years (*SD* = 10.50; Table 1) and included 643 people (225 females) with alcohol dependence and 323 controls (98 females). All sites had obtained ethical approval from local committees and written informed consent from all participants.

**Table 1:** Overview of sample’ demographic, substance use, and brain volume data in males and females with and without alcohol dependence.

	Mixed-effect models										
	HC		AD		Group (AD vs HC)		Sex (Males vs Females)		Group x Sex		Site^§^
	Males	Females	Males	Females	β (95% CI)	*p*	β (95% CI)	*p*	β (95% CI)	*p*	Var
**Demographic data**											
Sex, *N*	225	98	418	225	—	—	—	—	—	—	—
Age	30.34 (10.47)	29.48 (9.81)	34.21 (10.37)	32.68 (10.46)	5.83 (3.23, 8.42)	<0.001**	0.85 (−1.26, 2.96)	0.430	0.44 (−2.08, 2.96)	0.732	0.36
Education	15.17 (2.95)	15.69 (2.81)	13.99 (2.36)	14.35 (2.57)	−1.89 (−2.64, −1.13)	<0.001**	−0.30 (−0.92, 0.33)	0.352	−0.08 (−0.83, 0.67)	0.829	0.11
Monthly standard alcohol drinks	28.62 (30.25)	16.31 (20.18)	189.28 (184.06)	107.36 (107.82)	213.21 (167.58, 258.83)	<0.001**	12.98 (−21.97, 47.94)	0.466	37.29 (−3.40, 77.98)	0.072	0.38
Monthly cigarettes	25.32 (91.71)	43.95 (102.48)	179.85 (235.57)	139.99 (232.00)	250.09 (152.66, 347.53)	<0.001**	−24.52 (−102.08, 53.04)	0.536	49.57 (−36.24, 135.39)	0.258	0.19
ICV (10^6^)	1.44 (0.25)	1.24 (0.23)	1.62 (0.21)	1.42 (0.17)	−0.04 (−0.08,0.01)	0.115	0.19 (0.15,0.22)	<0.001**	0.05 (0.01, 0.09)	0.027*	0.58
**Brain volume data**											
**Amygdala**											
*Total Amygdala*											
L	1932.39 (203.74)	1716.69 (178.94)	1854.82 (205.64)	1702.83 (184.81)	−12.39 (−65.86, 41.07)	0.650	112.65 (67.57, 157.73)	<0.001**^a^	−75.28 (−126.53, −24.03)	0.004**^e^	0.23
R	1974.81 (228.07)	1760.24 (166.47)	1879.85 (203.20)	1724.33 (186.60)	−6.77 (−61.07, 47.53)	0.807	94.61 (48.95, 140.27)	<0.001**^b^	−70.68 (−122.57, −18.79)	0.008**^f^	0.29
**Amygdala nuclei**											
*Basolateral*											
L	1180.71 (121.25)	1052.04 (110.41)	1152.55 (123.75)	1059.05 (110.78)	−5.28 (−37.14, 26.56)	0.745	64.97 (38.14, 91.81)	<0.001**^c^	−43.95 (−74.48, −13.42)	0.005**^g^	0.24
R	1219.10 (141.99)	1087.45 (103.80)	1176.61 (124.07)	1081.97 (112.22)	5.06 (−28.03, 38.15)	0.764	56.70 (28.89, 85.51)	<0.001**^d^	−43.89 (−75.52, −12.27)	0.007**^h^	0.30
*Central*											
L	50.42 (9.21)	42.85 (8.35)	47.79 (9.53)	43.09 (8.32)	−1.50 (−4.05, 1.05)	0.248	2.75 (0.58,4.91)	0.013*	−1.92 (−4.37, 0.55)	0.127	0.14
R	55.83 (9.56)	48.45 (9.14)	53.80 (10.26)	47.84 (8.62)	−1.57 (−4.28, 1.13)	0.255	2.41 (0.11, 4.70)	0.040*	−1.22 (−3.83, 1.39)	0.360	0.16
**Hippocampus**											
*Total Hippocampus*											
L	3725.37 (384.73)	3471.15 (315.30)	3533.81 (371.41)	3329.20 (335.27)	−159.55 (−251.63, −67.47)	0.001**^i^	58.20 (−19.07, 135.48)	0.140	−64.69 (−152.46, 23.09)	0.149	0.35
R	3763.22 (390.15)	3519.05 (316.66)	3620.16 (359.34)	3368.18 (323.11)	−162.91 (−53.91, 188.23)	0.000**^j^	41.19 (−35.74, 118.11)	0.294	−22.14 (−109.53, 65.24)	0.619	0.34
**Hippocampus subfields**											
*CA1*											
L	694.13 (85.18)	638.80 (73.65)	654.99 (79.62)	608.60 (71.64)	−28.53 (−49.05, −8.00)	0.006**^k^	17.19 (−0.05, 34.42)	0.051	−12.73 (−32.32, 6.87)	0.203	0.32
R	719.71 (88.03)	664.51 (72.51)	689.17 (85.25)	638.07 (73.69)	−30.07 (−51.62, −8.52)	0.006**^l^	12.84 (−5.30, 30.97)	0.165	−7.42 (−28.04, 13.21)	0.481	0.27
*CA3*											
L	231.40 (38.30)	213.25 (30.42)	221.70 (34.49)	211.90 (29.80)	−5.72 (−15.01, 3.57)	0.227	3.46 (−4.35, 11.27)	0.386	−6.64 (−15.53, 2.24)	0.143	0.28
R	248.74 (36.96)	229.58 (32.35)	240.62 (34.34)	222.52 (28.52)	−9.29 (−18.56, −0.03)	0.049*	1.84 (−5.96, 9.65)	0.644	0.76 (−8.12, 9.64)	0.866	0.24
*Subiculum*											
L	461.34 (49.18)	432.02 (41.73)	451.91 (52.99)	424.58 (47.45)	−20.08 (−33.53, −6.62)	0.003**^m^	5.95 (−5.42, 17.32)	0.305	−2.45 (−15.39, 10.50)	0.711	0.19
R	445.99 (49.66)	422.88 (43.02)	445.66 (47.65)	416.63 (45.03)	−17.30 (−30.23, −4.37)	0.009**^n^	−0.21 (−11.15, 10.75)	0.971	2.55 (−9.92, 15.02)	0.689	0.17
*Dentate Gyrus*											
L	895.69 (101.61)	853.42 (83.64)	862.21(136.58)	829.86 (89.14)	−29.86 (−60.66, 0.94)	0.057	9.63 (−16.31, 35.58)	0.467	−29.46 (−58.95, 0.02)	0.050*	0.26
R	930.16 (103.34)	870.90 (84.56)	930.16 (103.34)	889.53 (137.93)	−35.99 (−67.42, −4.56)	0.025*	6.45 (−20.10, 33.00)	0.634	−13.40 (−43.60, 16.80)	0.384	0.20
Total Grey Matter	645436.15 (70122.99)	588728.40 (62720.93)	628371.75 (65854.89)	580707.99 (58610.65)	−15066.02 (−24848.77, −5283.267)	0.003**^o^	5690.41 (−2478.317, 13859.14)	0.172	−10182.12 (−19462.61, −901.63)	0.032*	0.39
Total White Matter	508814.31 (53343.32)	446133.76 (43868.89)	489254.52 (56721.53)	436481.00 (48150.03)	−23583.17 (−35033.04, −12133.30)	<0.001**^p^	8626.786 (−9.06.11, 18159.68)	0.076	−11661.24 (−22485.76, −836.72)	0.035*	0.59
Cerebrospinal Fluid	985.34 (279.50)	946.35 (244.36)	1127.87 (240.10)	992.07 (209.16)	67.12 (0.69, 133.56)	0.048*	−9.19 (−65.28, 46.90)	0.748	21.49 (−42.37, 85.36)	0.509	0.16

*Note:* HC = Healthy controls, AD = Alcohol Dependent subjects, β = beta, CI = confidence interval, Var = variation, R = right, L = left, ICV = Intracranial volume, § Site-level variation estimated as an intraclass correlation (ICC). Values for age, education, alcohol use, tobacco use and ICV are mean (standard deviation). Sex differences in sex distribution measured with chi^2^ test: χ^2^ = 2.09, *p* = 0.148. * *p*(uncorrected) < .05, ** ***p*(FDR)** **<** **.05**. All effects remained significant when tobacco use was added as an additional covariate to the model.

*Results of the pairwise comparisons of significant interactions (only significant comparisons are reported):* ^a^ M > F (β = 75.00, *p* < 0.001, d = −0.08); ^b^ M > F (β *=* 59.27, *p* < 0.001, d = -0.05); ^c^ M > F (β = 43.00, *p* < 0.001, d *=* -0.08); ^d^ M > F (β = 34.76, *p* < 0.001, d = -0.05); ^e^ AD male > HC male (β = -87.68 *p* < 0.001, d = -0.14), HC male > HC female (β = 112.64, *p* < 0.001, d = -0.25); ^f^ AD male > HC male (β = -77.45, *p* < 0.001, d = -0.11), HC male > HC female (β = 94.61, *p* < 0.001, d = -0.18); ^g^ AD male *>* HC male (β = -49.24, *p* < 0.001, d = -0.14), HC male > HC female (β = 64.97, *p* < 0.001, *d* = -0.24); ^h^ AD male > HC male (β = -38.83, *p* = 0.004, d = -0.09, HC male > HC female (β = 56.70, *p* < 0.001, d = -0.18); ^i^ HC > AD (β = -191.89, *p* < 0.001, d = -0.12); ^j^ HC > AD (β = -173.99, *p* < 0.001, d = -0.11); ^k^ HC *>* AD (β = -34.89, *p* < 0.001, d = -0.11); ^l^ HC > AD (β = -33.78, *p* < 0.001, d = -0.11); ^m^ HC > AD (β = -21.30, *p* < 0.001, d = -0.13); ^n^ HC > AD *(*β = -16.02, *p* = 0.001, d = -0.10); ^o^ HC > AD (β = -20157.08, *p* < 0.001, d = -0.11); ^p^HC > AD (β = -29413.79, *p* < 0.001, d = -0.09).

### Structural MRI data acquisition and processing

Automated parcellation of subcortical regions using structural T1-weighted brain images was performed at all sites using the recon-all pipeline in FreeSurfer 5.3 (http://sufrer.nmr.mgh.harvard.edu/)^45,46^. We then used new FreeSurfer 6.0 algorithms (implemented within the development version: devel-20180612)^40,47^ to segment the bilateral total amygdala and its nuclei (i.e., anterior amygdaloid, cortico-amygdaloid transition area, lateral, basal, paralaminar, accessory basal, medial, central, and cortical) and the bilateral total hippocampus and its subfields (i.e., tail, subiculum, presubiculim, parasubiculum, CA1, CA3, CA4, fissure, granular cells layer of the dentate gyrus [GC-ML-DG], molecular layer, hippocampus-amygdala transition area [HATA] and fimbria). Volumes for the a priori hippocampus dentate gyrus and for the basolateral amygdala were obtained by summing up those of smaller subregional outputs from FreeSurfer (i.e., hippocampus GC-ML-DG and molecular layer; amygdala basal and lateral nuclei, respectively).

All imaging data underwent standardised quality checks to reduce variability across sites, detect outliers, and invalid data points (http://enigma.ini.usc.edu/protocols/imaging-protocols). Additional stringent quality checks were run and entailed visual inspection of all hippocampal and amygdala subregions (see acknowledgments) using standardised protocols in collaboration with ENIGMA-MDD hippocampal subfields project which were adapted to apply to the new FreeSurfer algorithm (https://osf.io/b3uhw/).

### Statistical Analysis

Chi-squared tests were run to test sex differences between groups. Linear mixed models were performed to examine group, sex, and group-by-sex differences for years of age, years of education, number of monthly standard alcohol drinks^1^, number of monthly cigarettes, and brain volumes. Linear mixed models were chosen as they statistically accommodate dependency between observations (i.e., data points) in nested designs where data are collected from distinct research sites^48^. *Site* was treated as a random effect to account for the systematic site-level variation in the dependent variables expected to occur from differences in MRI scanner types and sequences, imaging, and behavioural testing protocols. Intra-class correlation (ICC) measured the extent of variation explained by site-level differences^48^.

First, we examined effects of group, sex, and group-by-sex interactions on the volumes of a priori amygdala volumes (total, and basolateral, and central nuclei) and hippocampus volumes (total, CA1, CA3, dentate gyrus, and subiculum subfields), adjusting for major confounders (age, education, and intracranial volume). Sensitivity analyses using tobacco exposure as a covariate (number of cigarettes per month), were further run on a subsample where information on these variables was also available (488 people with alcohol dependence and 140 controls).

Second, we measured if monthly standard drinks predicted a priori amygdala volumes (total, and basolateral, and central nuclei) and hippocampus volumes (total, and dentate gyrus, CA1, and CA3 subfields), separately in males and females with alcohol dependence, accounting for age, education, and intracranial volume. We assured that the assumptions for linear mixed models were met, including normal distribution of random effects. Monthly standard alcohol drinks were square root transformed as they showed positive skewness (i.e., 2.03 before and 1.00 after transformation).

All statistical models described above were also run in exploratory tests on the volumes of additional amygdalar nuclei (i.e., accessory basal, anterior, cortical, cortico-amygdaloid transition area, medial, paralaminar), hippocampal subfields (i.e., CA4, fimbria, fissure, HATA, parasubiculum, presubiculum, tail), and total cortical grey matter, white matter, and cerebrospinal fluid volumes.

A false discovery rate (FDR)-corrected statistical threshold of *p*(FDR) < 0.05 was used to control for multiple comparisons^49^ and was applied separately for a priori and exploratory analyses. Significant main or interaction effects were interrogated with pairwise comparisons between the groups and/or sexes. In the text, we express the percentage (%) calculated as a % difference between the two observed means. Effect sizes were estimated for the significant *p*(FDR) < 0.05 group and group-by-sex effects using Cohen’s *d* and based on the marginal means predicted by the model. All statistical analyses were performed using STATA 15.1 (StataCorp; 2017).

## Results

Basic demographic information and brain volume data are provided in Table 1. The alcohol-dependent and control groups were matched by sex. The group comprising people with alcohol dependence was older, had fewer years of education, and had higher monthly alcohol standard drinks and cigarettes than the control group. Males in both the alcohol-dependent and control groups consumed more monthly standard alcohol drinks than females. The alcohol-dependent group also had significantly smaller total grey and white matter volumes than healthy controls.

### Amygdala volumes

#### Group, sex, and group-by-sex effects on the volumes of the amygdala and its nuclei

**Fig. 1:**
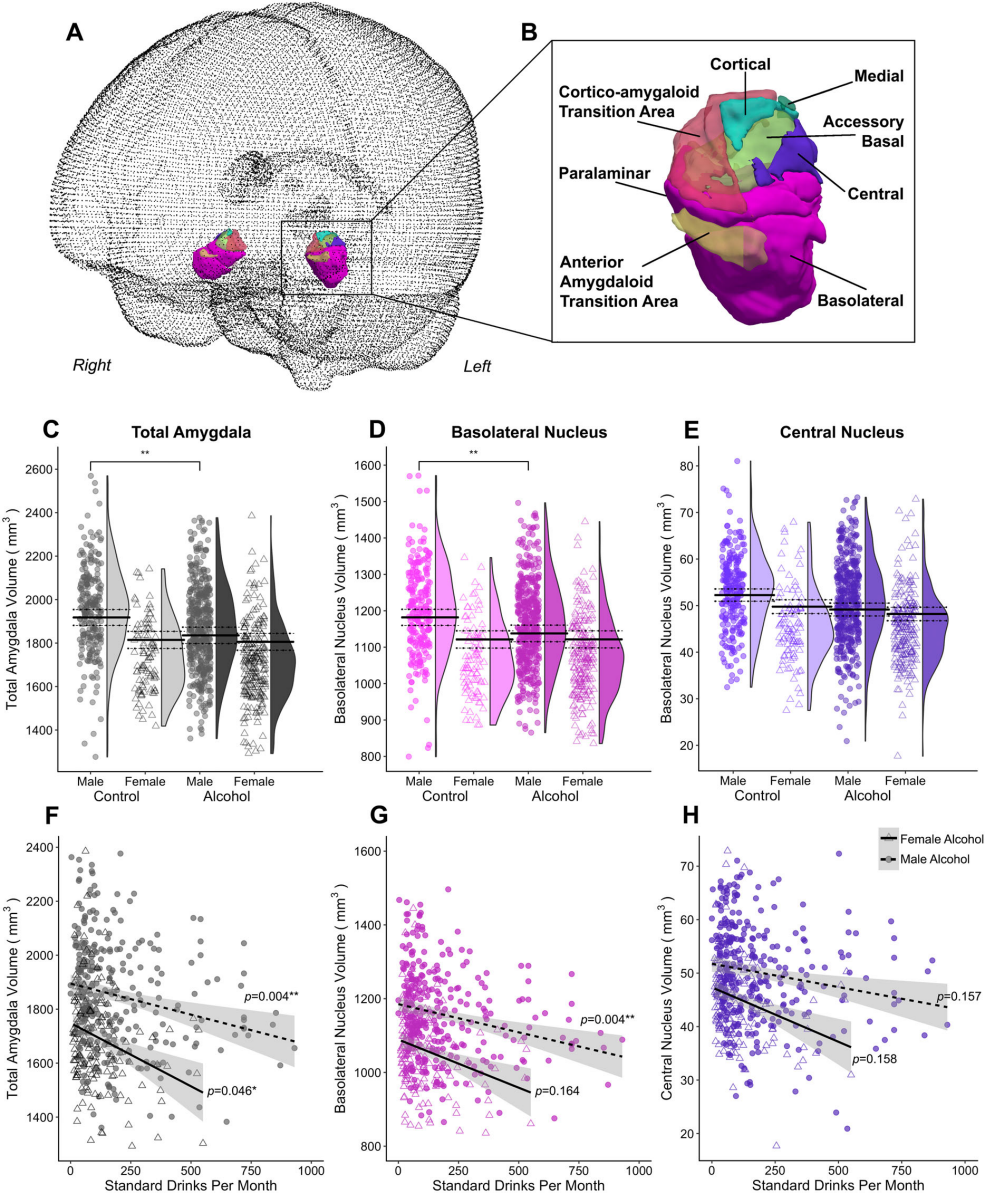
Overview of group by sex effects on the volume of a-priori amygdala region and amygdala nuclei. **A** Overview of glass brain section showing 3D rendering and **B** close up of 3D renders of all amygdala nuclei from an example participant. Plots of the a priori amygdala regions, comprising **C** total amygdala in greyscale, **D** basolateral nuclei in pink, and **E** central nuclei in purple containing individual data points and the probability density of the data stratified by group and sex (the group average of the estimated marginal means predicted by the models are indicated by the solid black line, with dotted lines indicating the standard error of the marginal means). The bottom panel shows regression plots for volume of the **F** total amygdala, **G** basolateral nuclei, and **H** central nuclei by alcohol use (standard alcohol drinks per month) in male and female alcohol-dependent participants, adjusted for intracranial volume, age, and education. Only the association in the male group for the total and basolateral amygdala remained significant after FDR correction. The left and right hemispheres for the nuclei have been collapsed. **p(FDR) < 05.

In the analysis of a priori amygdala volumes across group, sex, and group-by-sex (Table 1), there were no main effects of group, but there were significant main effects of sex and group-by-sex interactions for the bilateral total and basolateral amygdala (Table 1; accounting for age, education, intracranial volume, and tobacco use). Pairwise comparisons showed that alcohol-dependent males had significantly lower volumes of the total amygdala (5% smaller) and basolateral nucleus (3% smaller) than male controls (Fig. 1). There were no significant differences between females with alcohol dependence and female controls within the a priori amygdala nuclei. There were no significant group or group-by-sex effects within the central amygdala.

**Fig. 2:**
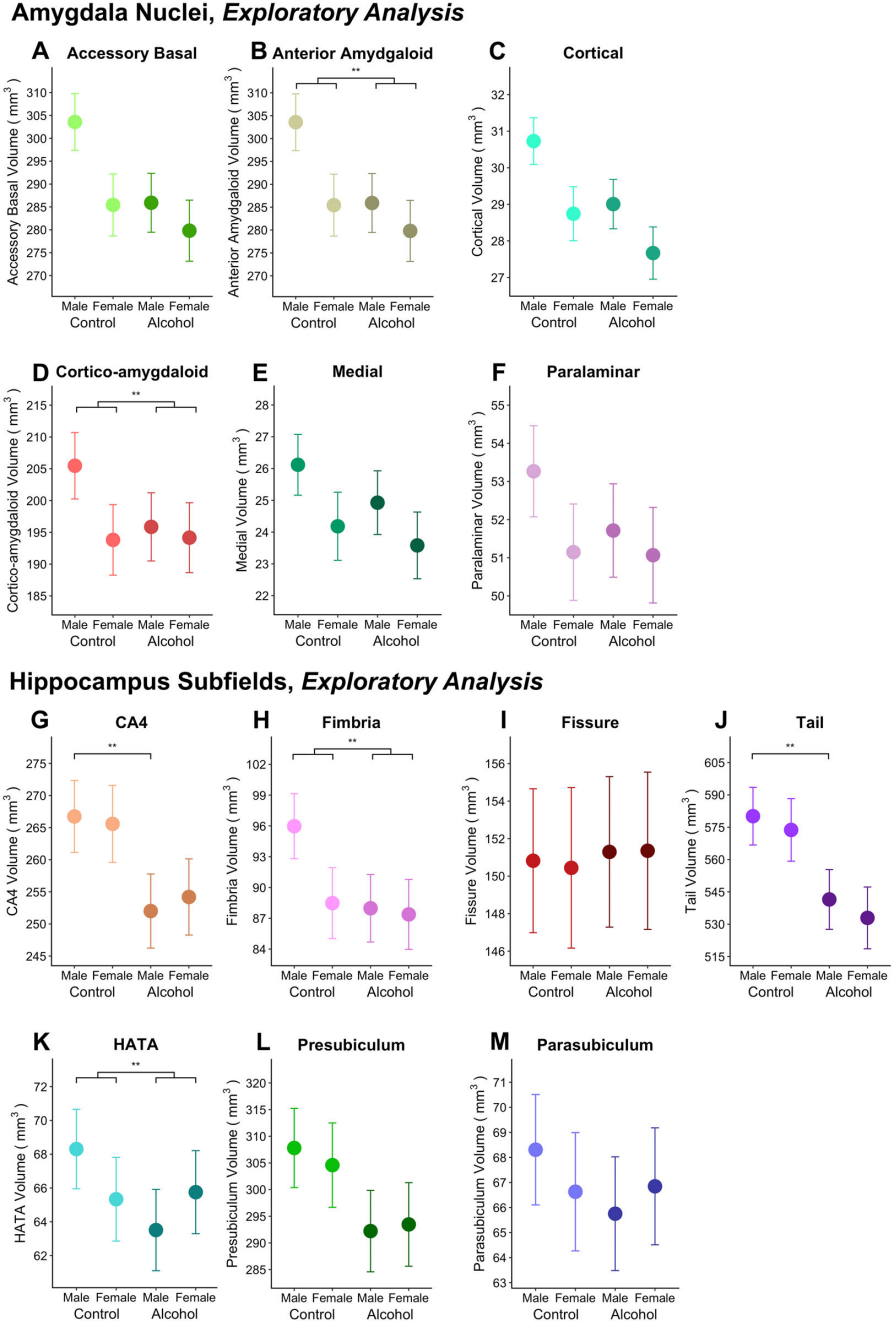
Overview of group by sex effects on the volume of exploratory amygdala nuclei and hippocampus subfields. Plots showing the estimated marginal means predicted by the model for the exploratory amygdala nuclei **A** accessory basal nucleus, **B** anterior amygdaloid transition area, **C** cortical nucleus, **D** cortico-amygdaloid transition area, **E** medial nucleus, and **F** paralaminar nucleus; and hippocampus subfields: **G** cornu ammonis 4 (CA4), **H** fimbria, **I** fissure, **J** tail, **K** hippocampal-amygdaloid tranisiton area (HATA), **L** presubiculum and **M** parasubiculum. The left and right hemispheres for the nuclei have been collapsed. Error bars represent the standard error of the marginal mean. **p(FDR) < 0.05.

Within select *exploratory* amygdala nuclei (i.e., accessory basal, anterior and cortico-amygdaloid transition; Table 2 and Fig. 2) there were significant main effects of sex (males larger than females) and significant group-by-sex interactions, whereby males (but not females) with alcohol dependence had significantly smaller volumes than male controls.

**Table 2:** Overview of mean (standard deviation) volumes of *exploratory* amygdala nuclei and hippocampal subfields across females and males with and without alcohol dependence, accounting for age, education and intra-cranial volume.

	HC		AD		Group (AD vs Controls)		Sex (Males vs Females)		Group x Sex		Site^§^
	Males (*n* = 225)	Females (*n* = 98)	Males (*n* = 418)	Females (*n* = 225)	β (95% CI)	*p*	β (95% CI)	*p*	β (95% CI)	*p*	Var
**Amygdala nuc**											
*Accessory basal nucleus*											
L	309.21 (39.38)	272.03 (32.75)	288.79 (38.90)	264.65 (33.83)	−5.12 (−15.37, 5.14)	0.328	20.09 (11.43,28.74)	<0.001**	−12.65 (−22.50, −2.81)	0.012**^a^	0.21
R	311.15 (40.48)	275.59 (31.07)	288.42 (36.73)	263.02 (33.66)	−6.55 (−16.61, 3.52)	0.202	16.36 (7.88, 24.84)	<0.001**	−11.19 (−20.84, −1.55)	0.023	0.24
*Anterior amygdaloid area*											
L	61.82 (9.47)	55.94 (9.00)	60.91 (8.65)	57.29 (8.14)	1.11 (−1.42, 3.64)	0.392	4.61 (2.47, 6.75)	<0.001**	−3.80 (−6.24, −1.36)	0.002**^b^	0.18
R	66.60 (10.45)	59.56 (7.65)	64.42 (9.14)	60.39 (8.44)	0.01 (−2.62, 2.63)	0.997	4.51 (2.29, 6.72)	<0.001**	−3.98 (−6.49, −1.47)	0.002*	0.25
*Cortical nucleus*											
L	27.05 (6.29)	23.24 (6.22)	26.50 (6.90)	22.92 (6.21)	−0.55 (−1.94, 0.85)	0.445	2.34 (1.15, 3.53)	<0.001**	−1.13 (−2.49, 0.23)	0.103	0.12
R	31.20 (29.70)	27.47 (3.95)	29.70 (5.50)	26.50 (4.60)	−1.61 (−3.06, -0.17)	0.029	1.71 (0.48, 2.95)	0.006**	−0.16 (−1.57, 1.25)	0.822	0.12
*Cortico-amygdaloid transition area*											
L	216.42 (30.90)	194.72 (22.52)	198.00 (25.87)	182.87 (23.73)	0.62 (−6.71, 7.94)	0.869	13.46 (7.30, 19.62)	<0.001**	−10.05 (−17.05, −3.05)	0.005**^c^	0.28
R	209.56 (28.02)	189.16 (19.23)	189.23 (25.08)	175.29 (23.62)	−0.29 (−7.07, 6.49)	0.933	9.96 (4.26, 15.65)	0.001**	−9.70 (−16.16, −3.22)	0.003**^d^	0.34
*Medial nucleus*											
L	26.56 (6.70)	22.09 (5.95)	24.10 (6.60)	21.33 (6.30)	−0.08 (−1.97, 1.08)	0.932	2.23 (0.64, 3.84)	0.006**	−1.16 (−2.98, 0.65)	0.210	0.18
R	27.05 (6.29)	23.24 (6.22)	26.50 (6.90)	22.92 (6.21)	−1.03 (−2.94, 0.87)	0.290	1.74 (0.12, 3.37)	0.035*	−0.06 (−1.91, 1.80)	0.949	0.14
*Paralaminar nucleus*											
L	55.89 (6.19)	50.15 (5.45)	52.55 (6.08)	47.92 (5.31)	−0.36 (−1.96, 1.23)	0.656	2.58 (1.24, 3.92)	<0.001**	−1.69 (−3.22, −0.17)	0.030	0.29
R	54.32 (6.51)	49.32 (4.92)	50.73 (5.61)	46.39 (5.07)	0.14 (−1.37, 1.66)	0.852	1.70 (0.44, 2.97)	0.009**	−1.23 (−2.67, 0.21)	0.095	0.37
**Hippocampus subfields**											
*CA4*											
L	268.00 (34.13)	249.78 (26.62)	257.05 (31.28)	247.44 (27.78)	−9.95 (−17.98, −1.92)	0.015*	1.47 (−5.27, 8.21)	0.668	−6.63 (−14.29, 1.03)	0.090	0.31
R	275.43 (32.92)	256.64 (28.29)	267.00 (30.68)	250.22 (25.12)	−12.92 (−20.92,−4.93)	0.002**^e^	1.04 (−5.69, 7.78)	0.761	0.02 (−7.64, 7.67)	0.996	0.26
*Hippocampal-amygdaloid*											
*Transition area*											
L	71.32 (12.84)	63.68 (10.33)	64.21 (11.27)	60.99 (10.30)	−0.89 (−3.97, 2.20)	0.573	3.81 (1.22, 6.41)	0.004**	−5.21 (−8.16, −2.26)	0.001**^f^	0.31
R	70.72 (12.61)	63.66 (9.02)	63.07 (10.99)	60.28 (10.47)	1.55 (−1.45, 4.55)	0.311	2.15 (−3.66, 4.68)	0.094	−5.11 (−7.98, −2.24)	<0.001**^g^	0.35
*Hippocampal fimbria*											
L	96.95 (19.72)	85.97 (16.98)	92.79 (20.92)	85.63 (17.19)	−2.42 (−7.82, 2.99)	0.380	6.00 (1.42, 10.57)	0.010**	−5.18 (−10.38, 0.02)	0.051*	0.19
R	93.58 (19.89)	79.67 (15.93)	86.83 (19.57)	81.02 (15.52)	0.12 (−5.11, 5.34)	0.966	8.86 (4.46, 13.27)	<0.001**	−8.49 (−10.38, 0.02)	0.001**^h^	0.23
*Hippocampal fissure*											
L	154.78 (24.94)	148.04 (27.65)	148.94 (26.64)	136.83 (25.80)	−0.76 (−8.21, 6.69)	0.842	0.51 (−5.83, 6.84)	0.876	0.85 (−6.37, 8.06)	0.818	0.14
R	152.73 (28.70)	143.09 (24.44)	146.82 (28.14)	134.36 (26.71)	2.02 (−5.25, 9.29)	0.586	0.52 (−5.63, 6.67)	0.869	−1.62 (−8.60, 5.38)	0.652	0.19
*Hippocampal tail*											
L	587.66 (79.02)	557.74 (67.70)	545.41 (70.27)	513.65 (67.18)	−39.57 (−60.24, −18.89)	<0.001**^i^	5.07 (−12.37, 22.49)	0.569	0.31 (−19.51, 20.12)	0.976	0.23
R	604.55 (82.97)	574.89 (67.16)	574.57 (69.95)	528.42 (68.00)	−42.39 (−63.27, −21.52)	<0.001**^j^	8.22 (−9.36, 25.80)	0.360	4.32 (−15.66, 24.30)	0.672	0.26
*Parasubiculum*											
L	73.35 (11.57)	67.01 (10.11)	64.37 (13.13)	59.63 (9.87)	−0.47 (−3.85, 2.90)	0.784	2.74 (−0.11, 5.60)	0.059	−3.51 (−6.76, −0.27)	0.034	0.22
R	70.55 (12.02)	65.57 (11.19)	62.65 (12.39)	57.83 (11.14)	0.53 (−2.84, 3.90)	0.759	0.74 (−2.09, 3.58)	0.607	−1.92 (−5.14, 1.30)	0.243	0.28
*Presubiculum*											
L	327.12 (41.72)	309.46 (35.72)	307.80 (42.76)	286.93 (34.72)	−13.73 (−24.52, −2.94)	0.013	4.98 (−4.10, 14.06)	0.282	−4.47 (−14.79, 5.85)	0.396	0.27
R	303.80 (40.48)	290.75 (34.96)	289.81 (37.40)	271.51 (33.59)	−9.09 (−19.14, 0.96)	0.076	1.59 (−6.85, 10.04)	0.711	−4.13 (−13.74, 5.49)	0.399	0.29

*Note:* HC = Healthy controls, AD = Alcohol Dependent subjects, β = beta, CI = confidence interval, Var = variation, R = right, L = left, § Site-level variation estimated as an intraclass correlation (ICC). * *p*(uncorrected) < .05, ** *p*(FDR) < 0.05.

*Results of the pairwise comparisons of significant interactions (only significant comparisons are reported):* all sex difference are males > females. ^a^ AD male > HC male (β = -17.51, *p* < 0.001, d = -0.14), AD male < AD female (β = 7.29, *p* = 0.023, d = 0.06), HC male > HC female (β = 20.08, *p* < 0.001, d = 0.22); ^b^AD male > HC ma le (β = -2.78, *p* = 0.006, d = -0.14), HC male > HC female (β = 4.64, *p* < 0.001, d = 0.05); ^c^ AD male > HC male (β = -9.19, *p* = 0.006, d = -0.09), HC male > HC female (β = 13.49, *p* *<* 0.001, d = 0.18); ^d^ AD male > HC male (β = -10.14, *p* < 0.001, d = -0.09), HC male > HC female (β = 10.17, *p* < 0.001, d = 0.13); ^e^ AD < HC (β = -12.91, *p* < 0.001, d = -0.11); ^f^ AD male > HC male (β = -6.09, *p* < 0.001, d = -0.15), HC male > HC female (β = 3.82, *p* = 0.004, d = 0.13); ^g^ AD male > HC male (β = -3.56, *p* = 0.003, d = 0.05), AD male < AD female (β = *-*2.96, *p* = 0.002, d = -0.07); ^h^ AD male > HC male (β = -8.38, *p* < 0.001, d = *-*0.14), HC male > HC female (β = 8.86, *p* < 0.001, d = 0.20); ^i^ AD < HC (β = *-*39.41, *p* < 0.001, d = -0.13); ^j^ AD < HC (β = -40.23, *p* < 0.001, d = -0.13).

#### Association between alcohol dosage and amygdala volumes in males and females

More monthly standard alcohol drinks predicted smaller total and basolateral amygdala volume in males, but not in females, with alcohol dependence (Fig. 1); after accounting for age, education, and intracranial volume. In females, there was a significant negative association between monthly standard alcohol drinks and volumes of the left central nucleus.

### Hippocampus volumes

#### Group, sex, and group-by-sex effects on the volumes of the hippocampus and its subfields

**Fig. 3:**
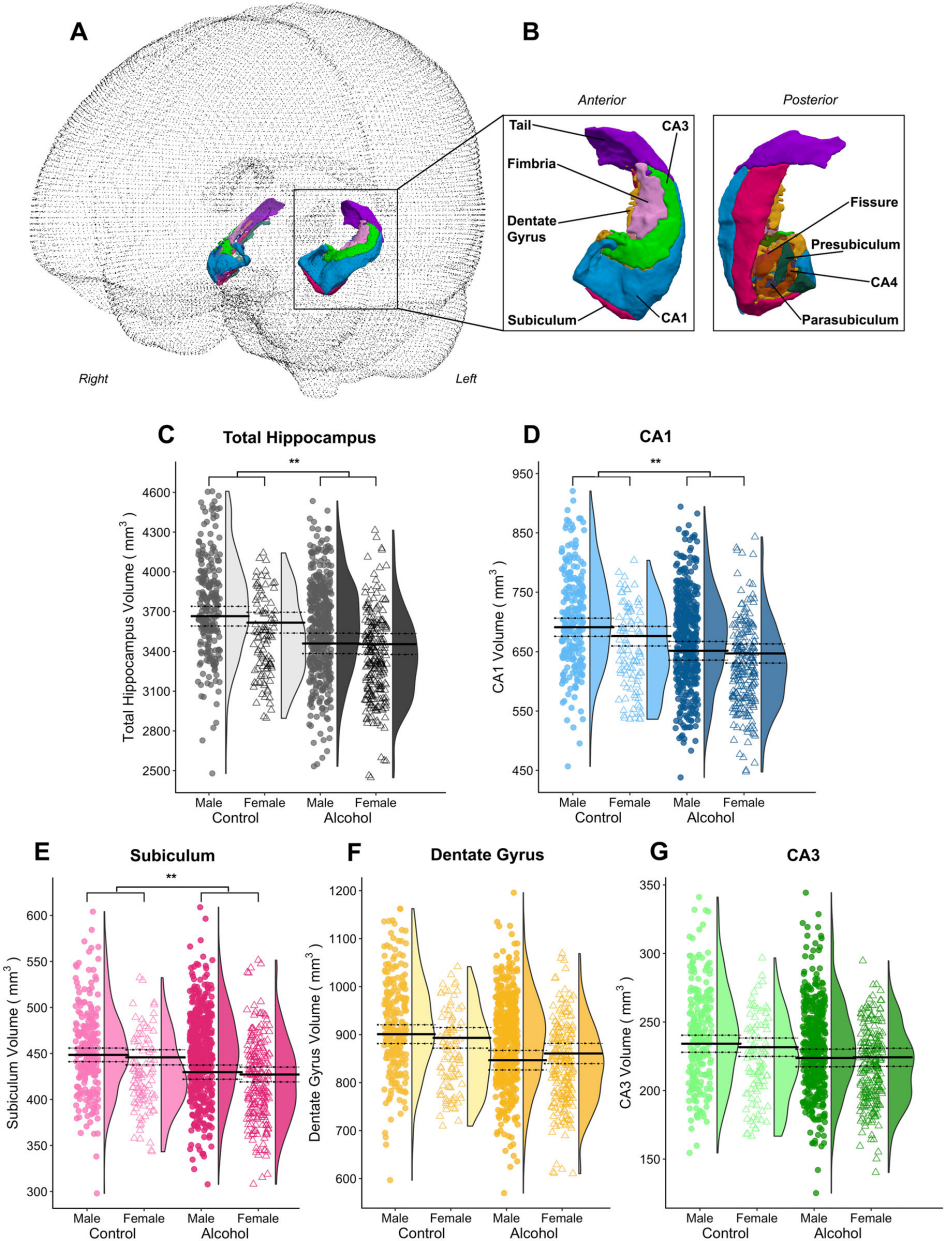
Overview of group by sex effects on the volume of a-priori hippocampual region and hippocampus subfields. **A** Overview of glass brain section showing 3D rendering and **B** close up of 3D renders of all hippocampal subfields from an example participant. Plots of the a priori **C** total hippocampus and **D** cornu ammonis 1 (CA1), **E** subiculum, **F** dentate gyrus, and **G** cornu ammonis 3 (CA3) subfields; containing individual data points and the probability density of the data stratified by group and sex (the group average of the estimated marginal means predicted by the models are indicated by the solid black line, with dotted lines indicating the standard error of the marginal means). The left and right hemispheres for the subfields have been collapsed. **p(FDR) < .05.

In the analysis of the a priori hippocampus regions, there were significant main effects of group within the bilateral total hippocampus and select subfields (CA1 and subiculum; Table 1), accounting for age, education, intracranial volume, and tobacco use. Pairwise analyses demonstrated 5% smaller volumes of the total hippocampus, 5% smaller CA1, and 3% smaller subiculum in the alcohol-dependent versus control group (Fig. 3). Specifically, the relative smaller volumes in males with alcohol dependence compared to male controls was comparable to that observed in alcohol-dependent females versus control females (e.g., 6% difference between males and 5% difference in females in the left hippocampus [*p*’s < 0.001]). We did not detect significant main effects of sex or interactions between group and sex for any a priori hippocampus subfields.

Within the *exploratory* subfields, there were significant main effects of group (Table 2. The alcohol dependent compared with the control group had significantly lower volumes of the right CA4 (3% smaller) and bilateral hippocampal tail (5% smaller); however, the significance of these effects dissipated when tobacco use was added as a covariate to these models. Within the *exploratory* HATA and fimbria hippocampal subfields, there were significant main effects of sex (males larger than females), and significant group-by-sex interactions whereby males with alcohol dependence compared to male controls had significantly lower bilateral HATA (11% smaller) and right fimbria (8% smaller) volumes, after accounting for age, education, ICV, and tobacco use (Table 2 and Fig. 2).

#### Association between alcohol dosage and hippocampus volumes in males and females

There was no association between monthly standard alcohol drinks and any of the hippocampal volumes in males or females.

### Sensitivity analysis on a priori amygdalar and hippocampal total and subregional volumes

In the alcohol-dependent group, males consumed significantly more monthly standard alcohol drinks than females (Table 1). Thus, we could not resolve whether dose-related lower volumes of the total and basolateral amygdala in males were driven by the higher drinking levels in males than females. To address this issue, we performed a *sensitivity analysis* (Supplementary Table 2). First, we matched the number of monthly standard drinks between men and women with alcohol dependence (β = 0.53, *95% CI* = [−0.15,1.21], *p* = 0.129) via removing alcohol-dependent males with the highest drinking levels (*n* = 186 males who consumed <280 monthly standard alcohol drinks). Then, we re-ran the statistical models that examined effects of group, sex, and group-by-sex on the volumes of a priori amygdalar and hippocampal volumes, and the association between alcohol dose and volumes in this sub-sample. The result of these analyses corroborated that main findings of a significant group-by-sex interaction within total and basolateral amygdala volumes (Supplementary Table 2). The negative interaction between monthly standard alcohol drinks and right total amygdala volume in males with alcohol dependence was also corroborated (β = −9.68, *95% CI* = [−16.67, −2.70], *p* = 0.007); however, the negative interactions between monthly standard alcohol drinks and volume of the left total amygdala or bilateral basolateral amygdala nuclei were not evident in the sensitivity subsample.

## Discussion

In this study, we demonstrated that alcohol dependence is associated with alterations of select subregions of the hippocampus and amygdala, some of which were similar and distinct between sexes. Our key findings were that males (but not females) with alcohol dependence had dose-dependent smaller volumes of the basolateral amygdala nucleus, and smaller volumes in exploratory subregions of the amygdala and hippocampus (i.e., cortico-amygdaloid transition, anterior, accessory basal, HATA, and fimbria). Additionally, alcohol dependence was associated with smaller volumes of total brain estimates of grey and white matter and within select hippocampal subfields (i.e., CA1, CA4, and subiculum), equally in men and women.

We report sex differences in the volumes of the total amygdala, select amygdala nuclei (i.e., basolateral, anterior amygdaloid area, accessory basal), and hippocampal subfields that connect with the amygdala (i.e., cortico-amygdaloid transition area and HATA)^50^. Our findings are partially consistent with those from previous work on alcohol dependence (i.e., smaller total amygdala and basolateral nucleus) where sex differences were not examined^28–30,38^. Lower amygdala volumes affected select amygdala nuclei that are implicated in behaviours characteristic of alcohol dependence, such as greater alcohol seeking^51^, higher alcohol craving and relapse risk^38^, and stress^52^ (i.e., anterior amygdaloid area, accessory basal nucleus). Thus, one could speculate that smaller volumes of select amygdala nuclei predate alcohol dependence in males. This may help explain why males are twice as likely to have alcohol dependence compared to females^28,30,38^. Alternatively, dose-dependent smaller volumes of the amygdala’s basolateral nucleus might reflect neuronal loss (e.g., apoptosis) due to the neurotoxic effects from the chronic exposure to ethanol, that is likely to occur in people with alcohol dependence^53^.

Within the hippocampus, we did not identify the predicted interactions between alcohol dependence and sex. We did, however, support our hypothesis and previous findings that people with alcohol dependence compared to controls have smaller hippocampal total volume^31–35^. This alteration may represent a shared neurobiological correlate as it was apparent in both males and females with alcohol dependence. We also replicated previous evidence that smaller total hippocampus volumes were driven by smaller volumes of select hippocampus subfields, including the CA1, CA4, and subiculum^38^. Lower hippocampal subfield volumes may reflect neuronal cell loss resulting from the neurotoxic effects of chronic alcohol exposure^20,22,23^, reduced hippocampal neurogenesis^17^, or represent a shared neurobiological vulnerability that predates alcohol dependence in both men and women. Volumetric reduction within the hippocampus may have implications for aberrant learning and memory processes characteristic of alcohol dependence^54^, particularly within the CA1 subfield which is implicated in memory retrieval and consolidation^27^.

We confirmed previous findings that alcohol dependence is associated with smaller global estimates of brain volume (grey and white matter)^37,55^. However, we failed to find any global or regional volumetric alterations specific to females with alcohol dependence. This finding supports evidence that a telescoping effect of alcohol dependence in females is not evident within studies of the general population^56^; however, it contrasted with our predictions and considerable evidence that females are more vulnerable to the neurotoxic effects of alcohol^3,37,44^. Sensitivity analyses corroborated our results in subsamples where males and females were matched by monthly standard drinks, and when analyses were re-run in males and females separately. We cannot determine whether lower amygdala volumes observed in alcohol-dependent males but not females (compared to their control counterparts) reflects either a (partly) distinct pathophysiology of alcohol dependence in different sexes, or phenotype differences in our sample that may have caused smaller amygdala nuclei volumes in males, but not in females, with alcohol dependence. For example, the structural alterations to the amygdala observed in males (but not in females) with alcohol dependence compared to controls, may have been caused by variables such as a distinct history of alcohol and other substance use, sex hormones, sleep quality, negative life events, and/or stress that differ between males and females^5^, that were not available in this sample. Likewise, a possible explanation for null results in females with alcohol dependence compared to female controls, is that only a subgroup of women with a high level of stress may be vulnerable to developing alterations of select amygdala and hippocampus subregions, as shown in animal models^14,57^. Indeed, our sample may have had heterogeneous levels of stress and related factors, as has been reported in previously examined samples of women with alcohol dependence (e.g., early life trauma, abuse)^14^. However, we cannot relate our data to stress levels as these were not available.

### Limitations

This study was cross-sectional, and we cannot exclude the possibility that the smaller volumes observed predated the onset of alcohol dependence. Longitudinal studies will be necessary to track whether alterations to hippocampus and amygdala subregional structure reflect trajectories before or during alcohol dependence or following its recovery. We had no data on mental health symptoms and limited data on substance use due to the heterogeneous testing protocols between sites. However, this was mitigated by screening for psychiatric comorbidities and controlling for the role of tobacco use and education. In addition, the new FreeSurfer segmentation algorithm has not been extensively validated; in response, we performed extensive visual quality assurance of all images (https://osf.io/wu78p/). A further strength of our study was that the large aggregated sample allowed adequate power to detect subtle effects of alcohol dependence and its interaction with sex.

## Conclusions

Alcohol dependence was associated with lower volumes within select hippocampus subfields in both males and females and within distinct amygdala nuclei in males only and in a dose-dependent fashion. Thus, we believe the systematic assessment of sex differences in alcohol dependence is warranted, using a subregion-specific approach and longitudinal designs, to track over time the neurobiological mechanisms that predate and follow alcohol dependence’ onset, recovery, and relapse in both males and females. This knowledge will be crucial to advance current neuroscientific theories of addiction and to ultimately inform personalised treatment targets.

## Supplementary information

Supplementary Material

## Data Availability

The datasets generated during and/or analysed during the current study are available from the corresponding author on reasonable request.

## Footnotes

1 Standard drinks were defined according to the National Institute on Alcohol Abuse and Alcoholism (NIAAA) criteria (https://www.niaaa.nih.gov/what-standard-drink).
