# Supplementary material for: Sex differences in the neuroanatomy of alcohol dependence: hippocampus and amygdala subregions in a sample of 966 people from the ENIGMA Addiction Working Group
Source: Transl Psychiatry. 2021 Mar 4;11:156. doi: 10.1038/s41398-021-01204-1 (PMC7933136; doi:10.1038/s41398-021-01204-1)
Supplement: Supplementary file 1 — Supplementary Material [file 41398_2021_1204_MOESM1_ESM.docx]

**Similarities and differences in hippocampus and amygdala subregions between males and females with alcohol dependence.**

Sally Grace^1^, Maria Gloria Rossetti^2,3^, Nicholas Allen^4^, Albert Batalla^2^, Marcella Bellani^2^, Paolo Brambilla^5,6^, Yann Chye^7^, Janna Cousijn^9^, Anneke Goudriaan^9^, Robert Hester^10^, Kent Hutchison^11^, Izelle Labuschagne^1^, Chiang-shan Ray Li^15^, Reza Momenan^12^, Rocio Martin-Santos^13^, Peter Rendell^1^, Nadia Solowij^14^, Rajita Sinha^15^, Lianne Schmaal^15,17^, Zsuzsika Sjoerds^18^, Chao Suo^7^, Gill Terrett^1^, Ruth van Holst^9^, Dick Veltman^8^, Murat Yücel^7^, Paul Thompson^19^, Patricia Conrod^8^, Scott Mackey^20^, Hugh Garavan^20^, & Valentina Lorenzetti^1,21,22^*

**SUPPLEMENTARY MATERIAL**

*

*

**Supplementary Table 1. Assessment instruments by imaging site**

|  | **Site 1** | **Site 2** | **Site 3** | **Site 4** | **Site 5** | **Site 6** | **Site 7** | **Site 8** | **Site 9** | **Site 10** |
| --- | --- | --- | --- | --- | --- | --- | --- | --- | --- | --- |
| *N* | 364 | 141 | 116 | 134 | 59 | 34 | 40 | 25 | 18 | 35 |
| Gender (F/M) | 137/227 | 54/87 | 29/87 | 44/90 | 24/35 | 0/34 | 15/25 | 0/25 | 2/16 | 18/17 |
| Site Location | University of New Mexico, Albuquerque | University of New Mexico, Albuquerque | Yale University, New Haven | National Institutes of Health Clinical Center, Bethesda | University of Amsterdam, Amsterdam | University of Amsterdam, Amsterdam | University of Amsterdam, Amsterdam | University of Barcelona, Barcelona | University of Wollongong, Wollongong | Monash University, Melbourne |
| **Inclusion criteria**  **(Alcohol)** | Alcohol dependence (DSM-IV; APA, 1994) | >/= 5 drinks/ per occasion (men)  >/= 4 drinks/ per occasion (women)  >/= five times in the past month. | Alcohol dependence (DSM-IV; APA, 1994) | Alcohol dependence  (DSM-IV; APA, 1994) | Alcohol dependence (DSM-IV; APA, 1994) | Alcohol abuse or dependence,  alcohol abstinence >/= 2 weeks | - | - | - | - |
| **Exclusion criteria (AD and HC)** | | | | | | | | | | |
| Axis I psychiatric disorders measurement | SCID | SCID | SCID | SCID | MINI plus | CIDI | MINI | PRISM, medical history | SCID | SCID, medical history |
| Axis I or II exclusion | Lifetime bipolar or psychotic disorder, current major depressive episode | Any lifetime Axis I disorder | Current Axis I disorder | Lifetime/curreNt schizophrenia/bipolar/psychotic disorder. Current mood/Anxiety excluded only if on psychotropic medications | Current Axis I disorder | Lifetime schizophrenia/psychotic disorder. Current Manic/OCD/PTSD | Any lifetime Axis I disorder | Any lifetime/current Axis I and II disorders | Any lifetime/current Axis I disorder | Any lifetime/current Axis I disorder |
| Urine alcohol toxicology (MRI day) | Positive screens | Positive screens | Positive screens | Positive screens | Positive screens | Positive screens | Positive screens (alcohol, illicit) | Positive screens (alcohol, illicit) | Positive screens (alcohol, illicit) | Positive screens (alcohol, illicit) |
| Breath alcohol test (MRI day) | Positive | Positive | Positive | Positive | Positive | - | - | - | - | - |
| General MRI contraindication | √ | √ | √ | √ | √ | √ | √ | √ | √ | √ |
| Other substances, Current abuse or dependence | √ | √ | √ | - | √ | √ | √ | √ | √ | - |
| Use of psychoactive medication | √ | - | - | - | - | - | √ | √ | √ | √ |
| Others | Severe alcohol withdrawal | Severe alcohol withdrawal |  | IQ < 80 | Use of psychoactive medication | Age< 18 years, - IQ < 80 |  | Left-handedness, female | Left-handedness |  |
| **Scanner parameters, 3T MRI scans** | | | | | | | | | | |
| Voxel size, mm^3^ | 1x1x1 | 1x1x1 | 1x1x1 | 0.9x0.9x2 | 1x1x1 | 1x1x1 | 1x1x1.2 | 1x1x1.1 | 1x1x1.1 | 1x1x1.1 |
| MR sequence | MPRAGE | MPRAGE | MPRAGE | MPRAGE | Gradient Echo | Gradient Echo | Turbo Field Echo | Spoiled Gradient Recalled Echo | Spoiled Gradient Recalled Echo | MPRAGE |
| Echo | 5-echo multi-echo | 5-echo multi-echo |  |  |  |  |  |  |  |  |
| TR, ms | 2350ms | 2350ms | 2350ms | 100ms | 9ms | 9ms | 9.6ms | 6.4ms | 6.4ms | 1900ms |
| TE, ms | 1.64, 3.5, 5.36, 7.22 & 9.08ms | 1.64, 3.5, 5.36, 7.22 & 9.08ms | 3.34ms | 12ms | 3.6ms | 3.6ms | 4.6ms | 2.9ms | 2.9ms | 2.15ms |
| Flip angle | 7° | 7° | 7° | 6° | 8° | 8° | 8° | 8° | 8° | 12° |
| Martrix | 256x256x176 | 256x256x176 | 256x256x176 | 256x256x124 | 256x231x170 | 256x256x170 | 256x256x182 | 256x256x180 | 256x256x180 | 256x256x176 |
| Manufacturer, model | 3.0T Siemens Trio | 3.0T Siemens Trio | 3.0T Siemens Trio | 1.5 T GE magnet | 3.0T Phillips Intera | 3.0T Phillips Intera | 3.0T Phillips Intera | 3.0T Siemens Trio | 3.0T Phillips Intera | 3.0T Siemens Trio |
| **Treatment seeking** | outpatients (with & without treatment seeking) | outpatients (with & without treatment seeking) | inpatients | inpatients | outpatients | outpatients (treatment seeking) | - | - | - | - |
| **Substance use measures** | |  |  |  |  |  |  |  |  |  |
| Pre-MRI abstinence | 24 h | 24 h | 11-17 days (mean:15 days) | 24 h | 24 h | ≥ 15 days (mean:18 days) |  |  |  |  |
| Severity of AUD | AUDIT, TLFB | AUDIT |  | AUDIT/TLFB | AUDIT | AUDIT |  |  |  |  |
| Standard drinks/month, *N* | √ | √ | √ | √ | √ | √ | √ | √ | √ | √ |
| Cigarettes/month, *N* | √ | √ | √ | √ | √ | √ | - | √ | √ | √ |

*Note:* AD= Alcohol-dependent subjects; HC= Healthy controls; ; SCID = Structured Clinical Interview for DSM Disorders (Spitzer et al. 1994; First et al. 2001); MINI = Mini Neuropsychiatry International Interview (Lecrubier et al. 1997; Sheehan et al. 1997; Swift et al. 1998); Inter- national Diagnostic interview (CIDI; Robins et al. 1988); PRISM = Psychiatric Research Interview for Substance and Mental Disorders (http://www.columbia.edu/~dsh2/prism/, Hasin et al. 1996); AUDIT = Alcohol Use Disorders Identification Test (Saunders et al. 1993); TLFB = Time-line Follow-back (Sobell and Sobell, 1992).

**Supplementary Table 2. Overview of sample demographic, substance use and brain volume data in male and females with and without alcohol dependence in the *sensitivity subsample* where males and females with alcohol dependence were matched on monthly standard alcohol drinks (*N* = 705).**

|  | |  | | |  | | Mixed-effect models (adjusted for age, education and ICV) | | | | | | |
| --- | --- | --- | --- | --- | --- | --- | --- | --- | --- | --- | --- | --- | --- |
|  | | HC | | | AD | | *Group*  *(AUD vs HC)* | | *Sex (Males vs Females)* | | *Group x Sex* | | Site^§^ |
|  |  | *Males* | | *Females* | *Males* | *Females* | β (95% CI) | *p* | β (95% CI) | *p* | β (95% CI) | *p* | Var |
| **Demographic data** | | | |  |  |  |  |  |  |  |  |  |  |
| Sex, *N* | | 225 | | 98 | 232 | 225 |  |  |  |  |  |  |  |
| Age | | 30.34(10.47) | | 29.48 (9.81) | 36.24(10.68) | 32.68(10.46) | 5.99(2.99, 8.99) | **<0.001^a^** | 1.03(-1.14, 3.20) | 0.353 | 1.42(-1.27, 4.11) | 0.301 | 0.37 |
| Education | | 15.17(2.95) | | 15.69(2.81) | 13.82(2.18) | 14.35(2.57) | -1.71(-2.53, -0.88) | **<0.001^b^** | -0.29(-0.92, 0.34) | 0.368 | -0.16(-0.94, 0.62) | 0.683 | 0.11 |
| Monthly standard alcohol drinks | | | 28.62(30.25) | 16.31(20.16) | 107.84(65.72) | 107.36(107.82) | 112.50(85.08, 139.92) | **<0.001^c^** | 14.01(-6.14, 34.16) | 0.173 | -14.89(-38.59, 8.82) | 0.218 | 0.22 |
| Monthly cigarettes | | 25.32(91.71) | | 43.95(102.48) | 145.61(223.25) | 139.99(232.00) | 239.54(144.91, 334.18) | **<0.001^d^** | -21.85(-97.43, 53.72) | 0.571 | 34.72(-49.62, 119.07) | 0.420 | 0.16 |
| ICV ^(10^6)^ | | 1.44(0.25) | | 1.24(0.23) | 1.64(0.20) | 1.42(0.17) | -0.03(-0.08, 0.02) | 0.215 | 0.18(0.15, 0.22) | **<0.001^e^** | 0.05(0.00, 0.09) | 0.035* | 0.58 |
| **Brain volume data** | | | |  |  |  |  |  |  |  |  |  |  |
| **Amygdala** |  |  | |  |  |  |  |  |  |  |  |  |  |
| *Total Amygdala* | L | 1932.39(203.74) | | 1716.69(178.94) | 1865.10(212.36) | 1702.83(184.81) | -19.45(-78.88, 39.97) | 0.521 | 108.40(63.02, 153.79) | **<0.001**** | -67.31(-120.38, -14.24) | **0.013**^f^** | 0.29 |
|  | R | 1974.81(228.07) | | 1760.24(166.47) | 1877.64(210.68) | 1724.33(186.60) | -17.77(-79.04, 43.49) | 0.570 | 91.70(45.13, 138.28) | **<0.001**** | -69.59(-124.02, -15.16) | **0.012**^g^** | 0.33 |
| **Amygdala nuclei** | | | |  |  |  |  |  |  |  |  |  |  |
| *Basolateral* | L | 1180.70(121.25) | | 1052.04(110.41) | 1159.98(129.35) | 1059.05(110.78) | -8.69(-44.51, 27.13) | 0.634 | 64.79(37.42, 92.18) | **<0.001**** | -38.02(-70.09, -5.96) | 0.020*^h^ | 0.28 |
|  | R | 1219.10(141.99) | | 1087.45(103.80) | 1178.12(129.38) | 1081.97(112.22) | -4.51(-41.93, 32.92) | 0.813 | 56.44(27.98, 84.91) | **0.001**** | -41.34(-74.66, -8.02) | **0.001**^i^** | 0.31 |
| *Central* | L | 50.42(9.22) | | 42.85(8.35) | 48.17(9.49) | 43.09(8.32) | -2.27(-4.98, 0.44) | 0.101 | 2.17(0.07, 4.26) | 0.043* | -1.91(-4.36, 0.54) | 0.670 | 0.20 |
|  | R | 55.82(9.56) | | 48.45(9.14) | 53.75(10.65) | 47.84(8.62) | -1.44(-4.42, 1.54) | 0.344 | 2.06(-0.24, 4.36) | 0.376 | -1.27(-3.96, 1.42) | 0.755 | 0.19 |
| **Hippocampus** | | | |  |  |  |  |  |  |  |  |  |  |
| *Total Hippocampus* | L | 3725.37(384.73) | | 3471(315.30) | 3543.64(370.09) | 3329.20(335.27) | -165.68(-267.04, -64.31) | **0.001**^j^** | 48.68(-267.04, -64.31) | 0.213 | -42.29(-131.69, 125.19) | 0.213 | 0.35 |
|  | R | 3763.22(390.15) | | 3519.05(316.66) | 3618.93(361.96) | 3368.18(323.11) | -167.95(-270.50, -65.35) | **0.001**^k^** | 33.78(-43.81, 111.38) | 0.393 | -15.63(-106.28, 75.00) | 0.299 | 0.32 |
| **Hippocampus subfields** | | | |  |  |  |  |  |  |  |  |  |  |
| *CA1* | L | 719.71(88.03) | | 638.80(73.65) | 657.41(79.78) | 608.60(71.64) | -27.62(-50.46, -4.79) | 0.018*^l^ | 15.80(-1.49, 33.08) | 0.073 | -7.59(-27.81, 12.64) | 0.462 | 0.29 |
|  | R | 231.40(38.31) | | 664.51(72.51) | 688.05(85.73) | 638.07(73.69) | -35.23(-59.12, -11.34) | **0.004**^m^** | 11.77(-6.41, 29.95) | 0.204 | -7.21(-28.49, 14.06) | 0.506 | 0.28 |
| *CA3* | L | 248.74(36.96) | | 213.25(30.42) | 223.54(32.36) | 211.90(29.80) | -5.33(-15.45, 4.79) | 0.460 | 2.89(-4.78, 10.57) | 0.460 | -5.54(-13.52, 4.44) | 0.321 | 0.31 |
|  | R | 914.11(103.39) | | 229.58(32.35) | 241.43(33.43) | 222.52(28.52) | -10.19(-20.35, -0.02) | 0.049* | 1.28(-6.47, 9.02) | 0.746 | 1.89(-7.18, 10.97) | 0.682 | 0.26 |
| *Subiculum* | L | 461.34(49.18) | | 432.04(41.73) | 452.95(53.97) | 424.58(47.45) | -21.75(-36.58, -6.91) | **0.004**^n^** | 5.05(-6.32, 16.43) | 0.384 | -0.97(-14.30, 12.36) | 0.886 | 0.21 |
|  | R | 445.99(49.65) | | 422.88(43.02) | 445.40(47.01) | 416.63(45.03) | -17.34(-31.65, -3.03) | 0.018*^o^ | -1.31(-12.31, 9.69) | 0.815 | 1.61(-11.28, 14.50) | 0.807 | 0.20 |
| *Dentate Gyrus* | L | 914.11(103.39) | | 853.42(83.64) | 857.88(160.02) | 829.86(89.14) | -28.82(-64.55, 6.91) | 0.114 | 8.00(-19.26, 35.26) | 0.565 | -27.76(-59.64, 4.11) | 0.088 | 0.25 |
|  | R | 930.16(103.34) | | 870.88(84.56) | 881.62(163.27) | 841.67(83.24) | -38.74(-75.18, -2.31) | 0.037* | 5.36(-22.69, 33.40) | 0.708 | -16.15(-48.98, 16.67) | 0.335 | 0.19 |

*Note:* HC= Healthy controls, AD= Alcohol Dependent subjects, β = beta, CI = confidence interval, Var = variation, R=right, L=left, OFC = orbitofrontal cortex , GM = Grey matter, WM = White matter, CSF= cerebrospinal fluid, § Site-level variation estimated as an intraclass correlation (ICC). Values for age, education, alcohol use, tobacco use and ICV are mean (SD). Sex differences in sex distribution measured with chi^2^ test: χ^2^ = 27.84, *p* < 0.001. * *p*(uncorrected) < .05, **** *p*(FDR) < .05**

*Results of the pairwise comparisons of significant interactions (demographics):* ^a^ AD >HC (β =6.71, *p* <0.001), ^b^ AD < HC (β =-1.79, *p* <0.001), ^c^ AD> HC (β =105.06, *p* <0.001), ^d^ AD > HC (β =256.90, *p* <0.001), ^e^ male > female (β =0.21, *p* <0.001).

*Results of the pairwise comparisons of significant interactions (volumes; only significant comparisons are reported):* all sex difference are males > females (*p* < 0.05); ^f^ AD male > HC male (β =-86.76, *p*=0.001, d=-0.16), AD male < AD female (β =41.09, *p*=0.030, d=0.07), HC male > HC female (β =108.40, *p*<0.001, d=0.23); ^g^ AD male > HC male (β =-87.36, *p*=0.002, d=-0.14), HC male > HC female (β =109.48, *p*<0.001, d=-0.17); ^h^ AD male > HC male (β =-61.02, *p*=0.001, d=0.19), AD male < AD female (β =23.53, *p*=0.039, d=0.27), HC male > HC female (β =77.24, *p*=<0.001, d=0.29); ^h^ AD male > HC male (β =-46.71, *p*=0.004, d=-0.14), AD male > AD female (β =26.77, *p*=0.004, d=0.08), HC male > HC female (β =64.78, *p*<0.001, d=0.24); ^i^ AD male > HC male (β=-45.85, *p*=0.007, d=-0.12), HC male > HC female (β=56.44, *p*<0.001, d=0.18); ^j^HC>AD (β =-186.82, *p* <0.001, d =0.12); ^k^ HC>AD (β =-175.74, *p* <0.001, d=0.11); ^l^ HC>AD (β =--31.42, *p*=0.001, d=0.10); ^m^ HC>AD (β =-38.84, *p*<0.001, d=0.13); ^n^ HC>AD (β=-18.54, *p*=0.001, d=0.13); ^o^ HC>AD (β=-16.54, *p*=0.006, d=0.11)
